# Modifying quinoa protein for enhanced functional properties and digestibility: A review

**DOI:** 10.1016/j.crfs.2023.100604

**Published:** 2023-09-30

**Authors:** Hao Cui, Siqi Li, Debashree Roy, Qing Guo, Aiqian Ye

**Affiliations:** a Riddet Institute, Massey University, Private Bag 11 222, Palmerston North, 4442, New Zealand; b School of Food and Advanced Technology, Massey University, Private Bag 11 222, Palmerston North, 4442, New Zealand; c College of Food Science and Nutritional Engineering, China Agricultural University, Beijing, 100083, China

**Keywords:** Quinoa protein, Protein modification, Functional property, Digestibility

## Abstract

Quinoa (Chenopodium quinoa Willd.) is a pseudocereal plant that originally came from South America. The trend of consuming quinoa is propelled by its well‒balanced amino acid profile compared to that of other plants. In addition, its gluten‒free nature makes quinoa a promising diet option for celiac disease patients. Protein accounts for approximately 17% of the quinoa seed composition and quinoa protein possesses excellent quality. Quinoa protein is mainly composed of 11S globulins (37%) and 2S albumins (35%), both of which are stabilized by disulfide bonds. To date, the alkaline extraction method is the most commonly used method to extract quinoa protein. The functional properties and digestibility of quinoa protein can be improved with the help of various modification methods, and as a result, the application of quinoa protein will be extended. In this review, the extraction method, modification of functional properties and digestibility of quinoa protein are thoroughly discussed, providing insights into the application of quinoa protein in plant‒based foods.

## Highlights

• Quinoa is a pseudocereal with great potential in food application.
• Quinoa protein possesses superior nutritional profile and good digestibility.
• Loss of certain proteins during the extraction process under alkaline conditions.
• Improvement of functional properties by physical, chemical, and enzymatic modification.

## Graphical abstract

**Figure:**
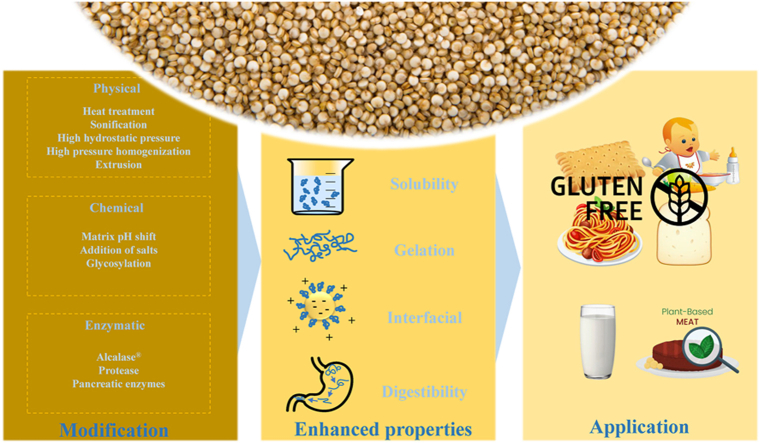

## 1 Introduction

Quinoa (Chenopodium quinoa Willd.), originating from Andean regions in South America, is classified as a pseudocereal. Quinoa belongs to the Amaranthaceae family, and it is a close relative of beets and amaranth (Vega-Gálvez et al., 2010). It was consumed as a domesticated staple food in Andean South America, although its leaves are also used as a potherb (Maughan et al., 2007). In recent decades, quinoa has gained considerable attention because of its potential to serve as a gluten‒free food (Alvarez-Jubete et al., 2010), its agricultural properties (Bazile et al., 2021) and its superior nutritional profile (Okon, 2021). Quinoa flour, when used in bread or extruded snacks, can serve as a high‒quality, gluten‒free option for individuals with celiac disease (Alvarez-Jubete et al., 2010; Muñoz-Pabon et al., 2022). Notably, the quinoa plant exhibits resistance to adverse agroecological conditions, including frost, soil salinity, and drought (Bazile et al., 2021; Hariadi et al., 2011; Jacobsen et al., 2005; Razzaghi et al., 2011). Moreover, quinoa seeds possess a superior nutritional profile compared to most traditional cereals owing to their essential amino acids (especially lysine, tryptophan, and cysteine), vitamins, minerals, fibre, and antioxidant compounds (Dakhili et al., 2019). In fact, compared to other plant proteins, the lysine content of quinoa protein is comparable to that of soybean and at least twice that of wheat, maize and rice (Abugoch James, 2009; Okon, 2021). Consequently, quinoa seeds are widely recognized as a ‘superfood’ and have gained popularity among customers with health needs.

Quinoa seeds are held on the plant in five-lobed perianths (Zhu, 2023). Quinoa seeds have a well‒balanced nutritional composition and are mainly made up of carbohydrates (60–74%), protein (13–17%), and lipids (2–10%) depending on the variety (Okon, 2021; Vega-Gálvez et al., 2010). Quinoa protein has attracted researchers’ attention not only because of its high content of nutrients but also because of its excellent quality. Quinoa protein contains complete essential amino acids necessary for human growth and metabolism (Vilcacundo and Hernández-Ledesma, 2017). Additionally, cooked quinoa protein was reported to have similar protein efficiency ratio (PER) values as casein (Abugoch James, 2009; Mahoney et al., 1975; Ranhotra et al., 1993). Moreover, short‒chain peptides released from quinoa protein have the potential to reduce the amount of free radicals during hydrolysis with Alcalase® (Aluko and Monu, 2003). Notably, removing saponins and cooking can effectively increase quinoa protein digestibility (Graf et al., 2015; Ruales and Nair, 1994).

The functional and digestive properties of quinoa protein play a significant role in potential food applications. Quinoa’s functional properties (such as solubility, water absorption capacity and oil absorption capacity, emulsifying properties, foaming properties and gelling properties) are considered in this review, as they are related to the texture of food products (Ge et al., 2020). In addition, proteins with high digestibility are favoured in the human diet because undigested proteins will be metabolized by colonic microflora and cause a long‒term detrimental effect on colonic health (Peled and Livney, 2021). Modification is a viable approach for enhancing the functional properties and digestibility of quinoa protein and can be categorized into physical modification, chemical modification, and enzymatic modification (Constantino and Garcia-Rojas, 2022).

To broaden the application of quinoa protein, it is essential to understand its physicochemical properties and digestive behaviour under different modification conditions. This review aims to summarize the effects of modifications on the functional properties and digestibility of quinoa protein. Furthermore, it provides an overview of the current quinoa protein applications and suggests potential future applications based on its properties.

## 2 Quinoa protein fractions

It is commonly agreed that quinoa protein consists of 11S globulins (37%), 2S albumins (35%), and a very minor portion of prolamins (0.5–7.0%) (Dakhili et al., 2019).

**Fig. 1:**
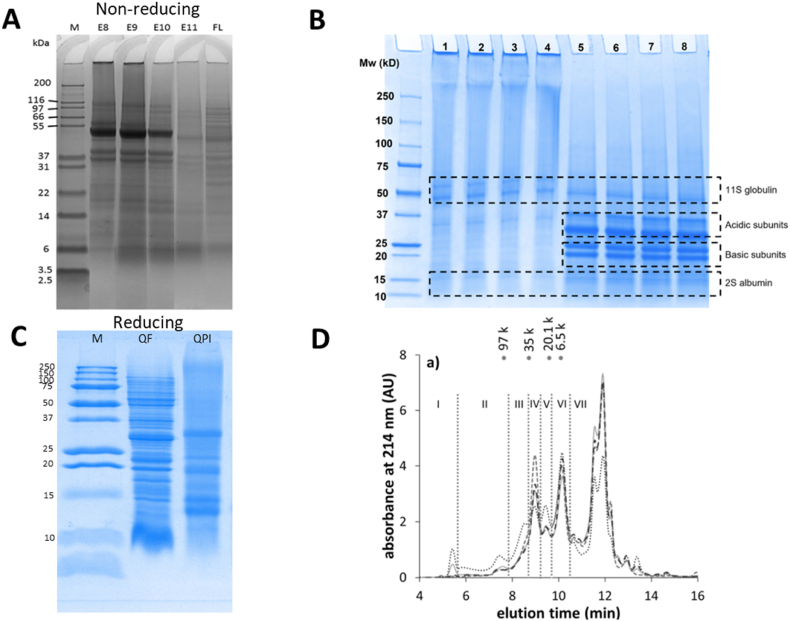
A: SDS-PAGE profile of the quinoa protein isolate QPIs extracted from pH 8–11 (noted as E8, E9, E10, E11) and defatted quinoa flour under non-reducing condition. Lane M: molecular weight marker; lane FL: defatted quinoa flour (Reprinted from Ruiz et al. (2016b)), with permission from Elsevier. B: SDS-PAGE profiles of untreated and HPH treated quinoa protein isolate QPI under non-reducing and reducing conditions. Lanes 1–4: non-reducing; Lanes 5–8: reducing. Lanes 1 and 5: non-treated; Lanes 2 and 6: 10 MPa; Lanes 3 and 7: 30 MPa; Lanes 4 and 8: 50 MPa. Mw: Molecular weight standards (Reprinted from Luo et al. (2022a)), with permission from Elsevier). The name of the proteins was labelled in correspondence of the bands. C: SDS-PAGE profile of wholegrain quinoa flour and quinoa protein isolate under reducing conditions. Lane M: molecular weight marker; Lane QF: wholegrain quinoa flour; Lane QPI: commercial quinoa protein isolate (protein content: 84%). D: SE-HPLC profiles of protein sequentially extracted (twofold, 10 min, 150 rpm) with water from wholemeal quinoa of cultivars Atlas (full grey line), Jessie (dotted black line), Pasto (dashed grey line) and Riobamba (dashed black line). Seven populations (I-VII) are distinguished. Molecular weights of markers (6.5, 20.1, 35 and 97 k) are indicated at the top of the SE-HPLC profiles. AU, arbitrary units (Reprinted from Van de Vondel et al. (2020), with permission from Elsevier).

11S globulins consist of six pairs of small basic and larger acidic polypeptides with molecular masses of 22–23 kDa and 32–39 kDa, respectively, linked by a single disulfide bond; these subunits (approximately 54 kDa) are stabilized by noncovalent interactions to form a hexamer (approximately 320 kDa) (Dakhili et al., 2019). Furthermore, 2S albumin in quinoa protein forms a heterodimer structure consisting of approximately 30–40 and 60–90 residues providing polypeptides with a molecular mass of approximately 8–9 kDa associated with two disulfide bonds (Dakhili et al., 2019). In addition to 11S globulin and 2S albumin, vicilin‒like 7S globulin was recently reported to be less abundant amount in the form of tetramers consisting of subunits of 16–66 kDa via noncovalent linkages (Quiroga et al., 2010). However, the abundance of the 7S globulin has not been extensively studied thus far. The fractions of proteins from quinoa seeds are illustrated via SDS‒PAGE and SE‒HPLC in Fig. 1.

Under nonreducing conditions, the majority of protein bands at approximately 55 kDa corresponded to 11S globulin (Fig. 1. A and B). These bands were reduced into acidic and basic subunits under reducing conditions (Fig. 1. B). Fig. 1C demonstrates the differences in protein components between quinoa flour and quinoa protein isolates, indicating a substantial loss of protein dry material during alkalinization and acid precipitation (Ruiz et al., 2016b). In Fig. 1. D, the proteins extracted with water via Osborne extractability contained a variety of proteins with different molecular weights, which denotes the complexity of the protein profile in quinoa seeds. The Osborne extraction procedures are based on difference in protein solubility, with albumins being water-extractable, globulins extractable in dilute salt, prolamins extractable in aqueous alcohol, and glutelins partially extractable in acid or base (Belitz et al., 2008).

Regarding the presence of prolamins in quinoa protein, there are differing opinions because studies have been based on extraction methodology (i.e., Osborne fractionation), ignoring the data from protein identification (Burrieza et al., 2020). On one hand, Burrieza et al. (2019) and Martínez et al. (2019) showed the absence of prolamins in quinoa seeds via genome and proteome analysis. On the other hand, the existence of prolamin‒like proteins in quinoa protein is contradictory to the fact that quinoa‒based foods are safe for celiac disease patients as confirmed by biochemical and immunochemical evidence (Burrieza et al., 2020; Peñas et al., 2014). This indicated the determined prolamin based on Osborne fractionation may not be relevant to immune response. However, it is noteworthy that some cultivars still had celiac‒toxic epitopes that were able to trigger immune responses in celiac disease patients (Zevallos et al., 2012). Therefore, careful consideration of the cultivar of raw materials is crucial for the application of quinoa protein in gluten‒free foods.

The structural and physicochemical properties of quinoa protein are dependent on the hierarchical structure at different length scales, including primary, secondary and higher‒order structures. For example, β-sheet or random coil can promote the formation of aggregation (Huang et al., 2022). Additionally, changes in the secondary structure induced the exposure of hydrophobic and hydrophilic groups, which may increase the solubility of quinoa protein isolate (Mir et al., 2021). Huang et al. (2022) reported that the dominant secondary structures in quinoa protein were β‒sheets (31.65%) and random coils (30.37%), followed by β‒turns (21.64%) and α‒helices (16.34%), which is in line with the results from Vera et al. (2019). However, cultivars, geographic varieties, and extraction methods collectively affect the proportions of secondary structures, and as a result, different results have been observed (Kaspchak et al., 2017; Shen et al., 2021; Wang et al., 2020b).

## 3 Quinoa protein extraction

Quinoa proteins are commonly extracted by alkaline extraction (pH 8.0–11.0) followed by acid precipitation at approximately isoelectric pH (pH 4.0–5.0) and subsequent drying steps (Ruiz et al., 2016b; Van de Vondel et al., 2020). Fig. 1. A and C demonstrate that quinoa flour contains more protein bands, indicating the loss of certain proteins during the extraction process, which is a limitation of the extraction process. The various solubilities of the protein fractions could partially explain the protein loss, since albumins are water‒extractable, globulins are dilute salt‒extractable, prolamins are aqueous alcohol‒extractable and glutelins are partially acid or base extractable (Belitz et al., 2008). Using Osborne extractability combined with mass spectrometry, Van de Vondel et al. (2020) identified the loss of proteins such as chloroplast and cytoplastic enzymes during alkaline extraction.

The extraction pH plays an essential role in the final protein composition (Fig. 1A). Fainter bands were observed at pH 11 under nonreducing condition. This could be explained by the occurrence of hydrolysed fractions with smaller sizes at extremely alkaline pH. On the one hand, as the extraction pH increased, more proteins were solubilized in the matrix and as a result, the protein yield increased (Ruiz et al., 2016b). On the other hand, a higher extraction pH induced changes in the physicochemical properties of quinoa protein, as investigated by Abugoch et al. (2008), who found that quinoa protein isolated at pH 11 possessed a lower solubility and a higher water‒imbibing capacity than quinoa protein isolated at pH 9. Moreover, higher extraction pH leads to decreased purity due to coprecipitation of nonprotein components into the extraction suspensions (Liu et al., 2023; Ruiz et al., 2016b). It is crucial to note that a higher extraction pH can cause protein denaturation and subsequently affect protein aggregation and functional properties (Van de Vondel et al., 2020).

**Fig. 2:**
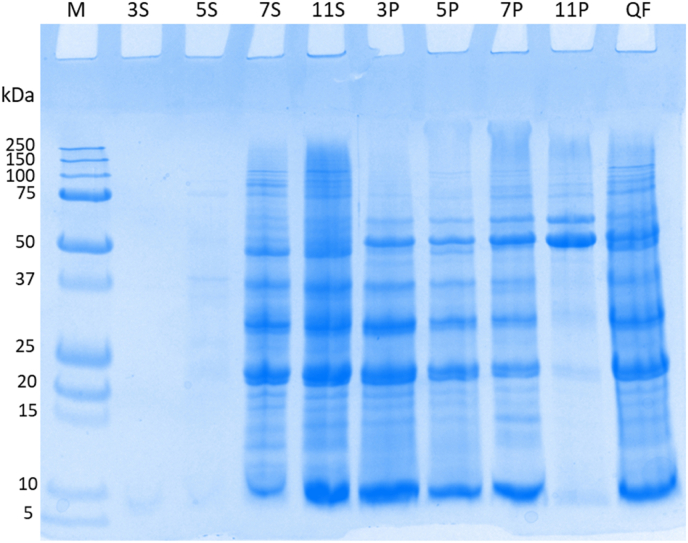
Reducing SDS-PAGE profile of extraction the supernatant and pellet fractions obtained from alkaline protein extraction from whole grain quinoa flour at different extraction pH after centrifugation at 6000*g*. M – marker. 3,5,7,9 – extraction pH of quinoa flour suspension (10% w/w). S – supernatant sample. P – pellet sample. QF – whole grain quinoa flour.

A clear difference in the protein composition under various pH conditions can be seen in Fig. 2. Whole grain quinoa flour was suspended in Milli‒Q water at different pH values (pH 3, 5, 7, 11) for 30 min with continuous stirring, followed by centrifugation to divide the suspension into supernatant and pellet. Most protein could be found in the pellets at pH 3 and pH 5 because the pH is close to the isoelectric point of quinoa 11S globulin (pH 4.5). When the pH increased from neutral to alkaline, a small number of proteins were still present in the pellets, especially proteins with a molecular weight of 50–75 kDa, indicating protein loss during alkaline extraction. In Fig. 2, the two protein bands between 50 and 75 kDa that appeared in all pellet samples are likely to be granule‒bound starch synthases (GBSS) with molecular weights of approximately 63 kDa and 56 kDa (López-Fernández et al., 2021). Protein loss via alkali extraction with isoelectric precipitation was also reported for quinoa (Ruiz et al., 2016b), pea (Tanger et al., 2020), lentil (Lee et al., 2007) and rice (Paraman et al., 2006).

Apart from Osborne extraction and traditional alkaline extraction, membrane technologies and ultrasound have also been incorporated to assist the extraction of quinoa protein (He et al., 2022b; Li et al., 2023; Navarro-Lisboa et al., 2017). Navarro-Lisboa et al. (2017) applied ceramic membrane ultrafiltration to obtain quinoa protein extracts from quinoa flour suspensions at pH 7.0 and pH 9.5. As a result, the protein concentration in the extracts reached 10.42 ± 0.21 mg/mL at pH 9.5, while 7.7 ± 0.1 mg/mL of recovered protein was obtained at pH 7.0. This indicated that ultrafiltration has potential application in the extraction of quinoa protein on a large scale; however, fouling problems involving protein‒membrane interactions and protein‒protein interactions need to be fixed to increase the effectiveness of the ultrafiltration process (Navarro-Lisboa et al., 2017). With the help of ultrasound treatment, Li et al. (2023) found a significantly improved extraction rate of quinoa protein compared to the traditional alkaline extraction process. The improved extractability might be attributed to the cavitation bubbles formed during ultrasound treatment affecting the medium in terms of mechanical, chemical, and thermal aspects. Ultrasound treatment generates cavitation and shear forces that can breakdown cells, enabling stronger solvent penetration into the quinoa protein and leading to an increase in its dissolution rate. However, excessive ultrasonic power may result in undesirable thermal effects that can denature the protein and lower its solubility (Li et al., 2023).

## 4 The functional properties of quinoa protein

Quinoa protein is gaining much attention for its nutritional profile and functional properties, which potentially promote its applications in the food industry (Abugoch et al., 2008; Elsohaimy et al., 2015; Ghumman et al., 2021; Mir et al., 2021). These functional properties, such as solubility, emulsifying properties, water absorption capacity (WAC), oil absorption capacity (OAC), foaming properties, and gelling properties, are key functional attributes for quinoa protein and are summarized in Table 1.

**Table 1:** Functional properties and digestibility of quinoa protein.^a^

Functional properties	Description	References
Solubility	Very low (25%) at acidic pH but reaches the maximum (75%) at around pH 10.	Elsohaimy et al. (2015)
Emulsifying properties	Low EAI (2.37–22.7 m^2^/g) but good ESI (34.70–123 min) for 1% (w/v) suspension.	Elsohaimy et al. (2015); Ghumman et al. (2021)
Water binding capability (g water/g protein)	2.0–3.9	Elsohaimy et al. (2015); Ghumman et al. (2021)
Oil binding capability (g oil/g protein)	1.88–3.32	Elsohaimy et al. (2015); Ghumman et al. (2021)
Foaming properties	FC at 50.8–60.2%, FS at 38.5–50.4% at 30 min.	Ghumman et al. (2021)
Gelling properties	LGC at 12% (w/v).	Wang et al. (2020a)
Digestibility	PDCAAS: 0.68	Scanlin and Lewis (2017)

a Abbreviations: EAI, emulsifying ability index. ESI, emulsifying stability index. FC, foaming capability. FS, foaming stability. LGC, the least gelation concentration. PDCAAS, protein digestibility corrected amino acid score.

In general, the functional properties of proteins can help create products with desired textures and other physical characteristics. For example, pH‒dependent solubility is closely related to the emulsification, gelation and foaming properties, and plays a pivotal role in determining the behaviour of protein‒rich food products (Idris et al., 2003; Kinsella and Melachouris, 1976). WAC and OAC show the ability to retain volatile components (e.g., flavours), and are also related to adhesion, film formation, fibre formation and viscosity. Gelling, induced by pH adjustment, heating conditions, and enzymatic processes, occurs at sufficiently high protein concentrations and involves the formation of three‒dimensional structures that retain water, flavours, sugars, and other food ingredients (Dakhili et al., 2019; Kinsella and Melachouris, 1976; Lingiardi et al., 2022). The functional properties of proteins influence the overall quality and sensory profile of food products (Ahmedna et al., 1999).

The solubility of quinoa protein is highly dependent on pH, being relatively low (25%) at acidic pH and reaching a maximum (75%) at approximately pH 10 (Elsohaimy et al., 2015). This trend of low solubility at acidic pH and high solubility at alkaline pH has also been reported for pea protein, soy protein, and chickpea protein (Ma et al., 2022; Zhao et al., 2020). Compared to dairy-based proteins, quinoa protein has inferior functional properties, especially low solubility and foaming stability and capacity, which is a common problem for plant-based proteins (Alrosan et al., 2022; Luo et al., 2022b). In terms of the emulsifying properties of quinoa protein, several studies have reported low emulsion activity index (EAI) but high emulsion stability index (ESI) for quinoa protein using the method proposed by Pearce and Kinsella (1978) (Elsohaimy et al., 2015; Ghumman et al., 2021). The EAI was 2.37 m^2^/g and 22.7 m^2^/g, while the ESI was 34.70 min and 123 min, respectively, for 1% (w/v) suspensions. The WAC and OAC of quinoa protein were observed to range from 2.0 to 3.9 g/g and 1.88–3.32 g/g, respectively. Variations in values reported by different researchers and challenges in comparing quinoa protein to other plant proteins arise due to the lack of standardized measurement methods. Differences in protein concentrations, mixing times, or centrifugation conditions can contribute to divergent results for WAC and OAC (Ma et al., 2022). According to Ghumman et al. (2021), quinoa protein showed high foaming capacity (FC) with good foaming stability (FS), with FC ranging from 50.8% to 60.2% and FS ranging from 38.5% to 50.4% at 30 min. Furthermore, Wang et al. (2020a) identified the least gelation concentration of quinoa protein at 12% (w/v). This value was comparable to that of soy protein (12%), higher than that of wheat gluten (8%), but lower than that of pea protein (14%) (Zhao et al., 2020).

In general, a variety of methods have been used to modify quinoa protein functional properties, including physical modifications (e.g., heat treatment, sonification, high hydrostatic pressure treatment (HHP), high‒pressure homogenization (HPH), extrusion), chemical modification (e.g., pH adjustment, divalent ions, Maillard reactions), and enzymatic modification (Table 2). In the following discussion, we will focus on the effects that these modifications have on the functional properties of quinoa protein.

**Table 2:** Modification of quinoa protein.^a^

Modification techniques	Modification treatment	Characterization techniques	Major findings	References
Physical modification	Heat treatment •Different temperatures from 25 to 121 °C with different time intervals from 5 to 30 min •Controlled heat-treatment (80–100 °C) at variable time (15–30 min) •Microwave heating at 560 W for 3 min •Steaming at 100 °C for 15 min •Boiling at 100 °C for 15 min •Baking at 160 °C for 5 min •81 ± 2 °C for 30 min	Intrinsic fluorescence UV-VIS FTIR CD SDS-PAGE XRD DSC	Solubility, WBC, OBC, EA, and ES were significantly improved after hydrothermal treatment. Moderate heat treatment resulted in the improvement of foaming capacity and foaming stability. Microwave heating and boiling improved solubility, emulsification and gelling properties while steaming and baking decreased such functional properties. Viscosity and elasticity of quinoa protein isolate gels increased after moderate heat treatment.	He et al. (2022a); Mir et al. (2021); Wang et al. (2020a); Luo et al. (2021)
	Sonification •HIUS at variable intervals from 5 to 35 min •HIUS at variable intervals and on-off pulses •HIUS at variable intervals for 5 and 15 min	Dynamic rheometer SDS-PAGE FT-IR Intrinsic fluorescence SEM CD UV-VIS DSC	Sonication significantly improved EA, ES, WBC and OBC compared to native quinoa protein isolate. EA, ES, WBC and OBC reached maximum for 25 min-HIUS-treated quinoa protein isolate. Sonication resulted in strong gelling behaviour and improved flow properties. Increased solubility was detected for HIUS treated quinoa protein isolate.	Mir et al. (2019); Vera et al. (2019); Luo et al. (2022b)
	HHP •250 and 600 MPa at room temperature for 15 min •HHP at room temperature at variable pressure form 100 MPa–600 MPa	SDS-PAGE FT-IR Rheometer CLSM SDS-PAGE	Quinoa protein isolate solubility increased to a small extent after HPP (600 MPa) treatment at pH 7 and pH 9. Viscosity and elasticity of quinoa protein isolate gels increased with the increase in pressure levels.	Luo et al., 2021, Luo et al., 2022b
	HPH •Pressure varied from 10 MPa to 50 MPa •Pressure varied from 30 MPa to 150 MPa	SDS-PAGE FT-IR SLS CLSM Rheometer Intrinsic fluorescence UV-VIS DSC	Enhanced emulsifying capacity, foaming capacity, solubility, and viscoelasticity. HPH treatments increased the solubility of quinoa protein. The peak value of EAI, ESI, FC, FS were obtained at 120 MPa. Shear stress and apparent viscosity of quinoa protein decreased as the pressure increased.	Luo et al. (2022a); Zhao et al. (2022)
	Extrusion •Semolina incorporated with quinoa protein isolate at variable levels •Quinoa flour	Colorimeter Rapid-Visco Analyzer Texture analyzer SEM SE-HPLC X-ray microtomography	Addition of quinoa protein isolate to produce pasta increased the optimal cooking time, water absorption, volume expansion, pasting temperature and firmness. Extrusion increased protein crosslinking and aggregation, and decreased protein solubility.	Gupta et al. (2021); Kuktaite et al. (2022)
Chemical modification	pH-shifting treatment •Acidic pH and basic pH (above 8.0) •pH 3.5 and 7.0	CD	Solubility shifted from low (less than 10%) to high (more than 70%) as the pH increased from acidic condition to alkaline condition. Quinoa protein isolate gel at pH 3.5 showed more stable to cooling than at pH 7.0.	Kaspchak et al. (2017); Elsohaimy et al. (2015)
	Presence of salts •The addition of CaCl_2_ or MgCl_2_ to quinoa protein isolate suspension •The addition of CaCl_2_ to quinoa protein concentrate suspension •The addition of NaCl and CaCl_2_ to quinoa protein isolate suspension	Rheometer SEM USANS SAXS SANS CLSM	The divalent ions resulted in stronger gels and crosslinking structure at pH 3.5, but had detrimental effects on gelation at pH 7.0. The incorporation of Ca^2+^ increased the quinoa protein concentrate suspension’s elastic behaviour. The gelation of quinoa protein isolate could occur at lower temperatures with increasing NaCl or CaCl_2_ concentration. Increasing the concentration of either NaCl or CaCl_2_ led to a greater gel strength.	Kaspchak et al. (2017); Quintero et al. (2022); Yang et al., 2022a, Yang et al., 2022b
	Glycosylation technology •Quinoa protein isolate grafted with mannose or xylose at variable levels	SDS-PAGE	Solubility, EA, ES, WAC and OAC were significantly improved, especially with 3 g of mannose.	Teng et al. (2021)
Enzymatic modification	•Limited Alcalase hydrolysis •Limited protease hydrolysis • Limited pancreatin hydrolysis	CLSM SDS-PAGE FT-IR Rheometer CSLM	Limited Alcalase hydrolysis could promote the thermally induced quinoa protein isolate gel strength, but the gel strength was strongly related to the hydrolysis time. Alcalase-hydrolysed quinoa protein showed higher solubility, emulsifying stability, and foaming capacity, but lower emulsifying activity index and foaming stability. The gel-forming ability and gel properties of acid-induced gels was affected by limited protease hydrolysis. Pancreatin hydrolysate of quinoa protein showed higher solubility, emulsifying and foaming activities but lower emulsifying and foaming stabilities than that of the control.	Wang et al. (2022); Aluko and Monu (2003); Galante et al. (2020); Daliri et al. (2021)

a Abbreviations: CD, circular dichroism. CLSM, confocal laser scanning microscopy. DSC, differential scanning calorimetry. EA, emulsion activity. ES, emulsion stability. FTIR, Fourier transform infra-red spectroscopy. HHP, high hydrostatic pressure. HIUS, high intensity ultrasound treatment. HPH, high pressure homogenization. OAC, oil absorption capacity. OBC, oil binding capacity. SANS, small-angle neutron scattering. SAXS, small-angle X-ray scattering. SDS-PAGE, sodium dodecyl sulfate–polyacrylamide gel electrophoresis. SE-HPLC, size exclusion-high-performance liquid chromatography. SEM, scanning electron microscopy. SLS, static light scattering. USANS, ultrasmall angle neutron scattering. UV-VIS, ultraviolet–visible spectroscopy. WAC, water absorption capacity. WBC, water binding capacity. XRD, X-ray diffraction.

### 4.1 Heat treatment

Heat treatment is the most common way to modify the functional properties of proteins. Heating leads to the denaturation or unfolding of proteins when the temperature reaches a certain level. Moreover, heating can alter the secondary, tertiary, and quaternary structures of polypeptide chains. Upon heating, these chains may expose embedded hydrophobic groups and aggregate through molecular interactions (Mahler et al., 2009).

He et al. (2022a) applied hydrothermal treatments at different temperatures for different intervals to quinoa protein isolates. Hydrothermal treatment significantly altered the secondary and tertiary structures and quaternary structures by unfolding with increasing particle size, which could be due to the denaturation of molecules and the formation of aggregates of quinoa protein. He et al. (2022a) found that the solubility of quinoa protein isolate reached a maximum while heating at 90 °C for 30 min, which was 33.4% higher than that of the native quinoa protein isolate but excessive temperatures (e.g., 100 °C and 121 °C) led to reduced solubility. A similar trend was also observed by Mir et al. (2021). In addition to the solubility, the water‒binding capacity (WBC), oil‒binding capacity (OBC), emulsifying ability (EA), and emulsifying stability (ES) of quinoa protein isolate were improved after heat treatments at 60–90 °C for 5–30 min, but treatments at 100 °C and 121 °C had negative effects on such properties. Similarly, Mir et al. (2021) found that moderate heat treatment conditions result in limited but favourable structural changes that play an essential role in the improvement of functional properties. Notably, Luo et al. (2021) revealed that moderate heat treatment increased the viscosity and elasticity of quinoa protein isolate gels, resulting in a stiffer gel with a compact network structure, where extensive intermolecular disulfide linking formed during heating may play an important role. Makinen et al. (2016) also observed the presence of disulfide cross‒linking in quinoa globulin aggregates at pH 6.5 and pH 8.5 after boiling for up to 15 min.

Wang et al. (2020a) compared the effects of different heating methods on the structure and functional properties of quinoa protein isolate. Among microwave heating, steaming, boiling, and baking, quinoa protein isolate treated with microwave heating and boiling exhibited better solubility, emulsification and gelling properties, while steaming and baking were detrimental to such functional properties. This phenomenon was attributed to the structural changes during the heating process, especially the aggregation of protein subunits, surface hydrophobicity and sulfhydryl content. Steaming and baking resulted in molecular aggregation and the formation of insoluble polymers (Wang et al., 2020a).

### 4.2 Sonication

High‒intensity ultrasound treatment (HIUS) is a novel and green approach to modify the native structure of proteins. Low‒intensity ultrasound has a frequency above 100 kHz with an intensity below 1 W/cm^2^, while high‒intensity ultrasound has a frequency ranging from 20 to 500 kHz with an intensity above 1 W/cm^2^ (Guimaraes et al., 2019). HIUS generates sound waves that create alternate compression in the medium, leading to the collapse of bubbles and the creation of cavitation; the imploding bubbles create increased temperature and pressure, so high shear energy waves accumulate in the cavitation zone (Arvanitoyannis et al., 2017). Therefore, HIUS can be used as a method to disrupt the noncovalent bonds (e.g., hydrophobic interactions and hydrogen bonding) that stabilize the structures of proteins, which can explain the decreased particle sizes, larger surface areas for water accessibility and higher solubility (Constantino and Garcia-Rojas, 2022; Luo et al., 2022b).

Vera et al. (2019) reported an increased solubility for HIUS‒treated quinoa protein extract because of conformational changes and soluble protein aggregation via disulfide bridges. Luo et al. (2022b) confirmed the increase in solubility of quinoa protein isolate after sonification as well. Additionally, HIUS‒treated quinoa protein isolate showed promising results with better gelling characteristics, emulsifying properties, and water and oil binding capacity (Mir et al., 2019). The improvement in gelling properties was proportional to the time of HIUS treatment. The enhanced functional properties could be associated with a stronger protein‒protein aggregation induced by HIUS and increased protein solubility.

### 4.3 High hydrostatic pressure treatment

High hydrostatic pressure treatment (HHP) has been widely used as a cold pasteurization technology that can cause reversible (e.g., dissociation of polymers) and nonreversible effects (e.g., unfolding, denaturation and gelation) (Constantino and Garcia-Rojas, 2022; Luo et al., 2022b). Therefore, it is an effective method to modify protein structures and properties.

Luo et al. (2022b) applied HHP treatment (up to 600 MPa) to quinoa protein isolate and SDS‒PAGE revealed that 11S globulins aggregated via disulphide bonds during HHP. The aggregation of 11S globulins also explained the results that free SH content and surface hydrophobicity decreased as pressure increased. The unfolding of proteins after HHP led to protein aggregates with large molecular weights and consequently decreased the surface hydrophobicity (Luo et al., 2022b). In addition, HHP caused the transformation of the secondary structure of quinoa protein from ordered (α‒helix and β‒turn) to disordered structure (β‒sheet and random coil). The aggregation and reassociation of protein structures under HHP treatment could explain the slightly increased solubility and reduced particle size of quinoa protein isolate, especially at pH 7 and pH 9 (Luo et al., 2022b).

In another study, these authors found that the gelling properties of quinoa protein isolate dispersions were improved by HHP (Luo et al., 2021). The viscosity and elasticity of quinoa protein isolate gels increased with increasing pressure level during the HHP treatment of quinoa protein isolate. Furthermore, confocal micrographs of quinoa protein isolate dispersions showed the interconnection that built a compact three‒dimensional protein network under pressure treatments (400–600 MPa), which was consistent with the better gelling properties of quinoa protein isolate dispersions after HHP. The unfolding and aggregation of 11S globulin through disulfide bonds as well as other possible noncovalent interactions (such as hydrophobic and electrostatic interactions, and hydrogen bonding) participated in the formation of quinoa protein isolate gels (Luo et al., 2021; Sun and Arntfield, 2012).

### 4.4 High pressure homogenization

High‒pressure homogenization (HPH) is a technology that is being well-established for use in the food research and industry. It is a relatively new technology with many potential applications, such as improving liquid food safety and extending shelf life without the detrimental effects of thermal processing. During the HPH process, the fluid sample flows through a narrow opening in the homogenizer valve. The pressure difference between the inlet and outlet of the fluid in the valve causes several physical effects to occur at the same time, including cavitation, turbulence, and shear (Dos Santos Aguilar et al., 2018). As a result, these phenomena alter the characteristics of the samples.

According to Luo et al. (2022a), HPH significantly improved the solubility, emulsifying capacity, foaming capacity, and gelling properties of quinoa protein isolate. The protein profile revealed by SDS‒PAGE and FTIR was not significantly altered by HPH treatment. However, protein unfolding was identified by the increased exposure of hydrophobic surfaces. HPH treatment decreased the quinoa protein particle size accompanied by a change from a bimodal to a monomodal distribution. Consequently, quinoa protein solubility was promoted, thereby improving functional properties (Luo et al., 2022a). In addition, CLSM revealed denser and homogeneous protein networks of quinoa protein isolate suspensions after HPH treatment, which was highly related to the enhanced gelling properties. Zhao et al. (2022) also confirmed the positive effects of HPH on the solubility, emulsifying properties, and foaming properties of quinoa protein isolate, and 120 MPa was the optimum condition. However, the shear stress and apparent viscosity of quinoa protein isolate gradually decreased as the pressure increased (Zhao et al., 2022). The decrease in shear stress may be due to the breakdown of protein molecule interactions and increased solubility caused by HPH treatment, while the decrease in apparent viscosity was possibly due to the disruption of chemical bonds and hydrophobic interactions among protein chains caused by cavitation resulting from HPH treatment (Shao et al., 2011; Wu et al., 2019; Zhao et al., 2022).

### 4.5 Extrusion

The extrusion process involves mechanical energy, pressure and heat. The raw material is pushed or “extruded” through a screw inside a barrel and then a die, which render the end product a specific shape (Ganjyal and Hanna, 2002). Extrusion technology can be divided into two categories based on the moisture rate: low (15–30% moisture) and high (up to 70%) moisture extrusion (Guyony et al., 2022). Both high and low moisture extrusion technology have been successfully applied to various plant proteins, such as high moisture extrusion for soy protein (Pietsch et al., 2019), pea protein (Osen et al., 2014), and peanut protein (Zhang et al., 2020), and low moisture extrusion for wheat gluten (Lin et al., 2022) and pea protein (Beck et al., 2017). However, to the best of our knowledge, few studies have applied extrusion cooking to modify quinoa protein.

According to Gupta et al. (2021), quinoa protein isolate‒incorporated extruded pasta showed increased optimal cooking time, swelling index, water absorption, and firmness but decreased whiteness index and viscosity, indicating that the quinoa protein isolate performed well during extrusion and has the potential to produce pasta with enhanced nutritional and functional properties. Kuktaite et al. (2022) found that extruding quinoa flour into extruded puffs facilitated quinoa protein crosslinking and aggregation and induced morphological changes because of extrusion and postprocessing heating. Scanning electron microscope (SEM) and X‒ray microtomography uncovered a compact and dense protein network stabilized by disulfide linkages in the extruded samples.

### 4.6 Matrix pH‒shifting treatment

Matrix pH where proteins are dissolved can induce changes in structural and functional properties of proteins. For example, at extreme alkaline conditions, denaturation and unfolding can occur, exposing sulfhydryl and hydrophobic groups, which could possibly induce new protein interactions (Nikbakht Nasrabadi et al., 2021). The solubility of quinoa protein is highly dependent on pH, where solubility was the lowest at acidic pH (close to pH 4.5) and reached a maximum at pH 11. This is because the isoelectric point of 11S quinoa globulin, the major protein in quinoa protein isolate, is approximately pH 4.5 to 5.0 (Shen et al., 2021), while the isoelectric point of 2S albumin was reported to be 3.4 by Yang et al. (2022a). The solubility of quinoa protein is influenced by the balance between hydrophilic and hydrophobic regions of the protein, as well as its interaction with the solvent. When the pH of the solvent is alkaline, the presence of negatively charged particles due to the ionization of certain chemical groups on the protein can improve the interaction between the protein and the solvent, increasing the solubility of the protein (Dakhili et al., 2019).

Another functional property that can be affected by pH is the gelling property. The gelling ability is largely affected by the pH level mainly because the aggregation of globulins is strongly influenced by pH, as is the effect on the solubility and secondary structure of quinoa proteins (Dakhili et al., 2019; Kaspchak et al., 2017; Makinen et al., 2016). In terms of secondary structure, the presence of an α‒helix structure led to a more elastic gel while the presence of a β‒sheet structure led to a harder gel (Su et al., 2015). Heat‒induced gelation of quinoa protein isolate occurs at acidic pH (3.5) and neutral pH (7.0), but the gels formed at pH 3.5 were more viscoelastic and denser than those formed at pH 7.0 (Kaspchak et al., 2017). This could be explained by two possible mechanisms: 1) The high protein solubility at pH 7.0 restrained gel formation, and 2) disulfide bonds of quinoa proteins remained intact and reduced the conformation flexibility of proteins; i.e., α‒helix, β‒sheet, β‒turn and random coil configurations were found in the secondary structure of QPI at pH 3.5, while at pH 7.0, α‒helix was found almost exclusively (Kaspchak et al., 2017; Makinen et al., 2016).

### 4.7 Presence of salts

According to Kaspchak et al. (2017), at pH 3.5, Ca^2+^ and Mg^2+^ ions significantly affected the gel formation process by inducing stronger gels with a fibrous gel network, while these ions had the opposite effect and made it more challenging to form a gel at pH 7.0. Similar findings were reported by Quintero et al. (2022), who found that the incorporation of Ca^2+^ and the decrease in pH close to the isoelectric point favoured the elastic behaviour of quinoa protein suspensions. Moreover, Yang et al. (2022b) investigated the heat‒induced gelation of quinoa protein isolate in the presence of salt (Na^+^ and Ca^2+^) and these authors found that the gelation of quinoa protein isolate could occur at lower temperatures with increasing NaCl or CaCl_2_ concentration, and increasing the concentration of either NaCl or CaCl_2_ led to a greater gel strength. Overall, the promoted gelation behaviour by the presence of salts could be attributed to the diminishing effect of salts on electrostatic repulsions, enhancing the attractive interactions between protein molecules to form stronger gels (Ikeda et al., 1999). As a result, protein aggregation occurred and gelation occurred when the protein concentration was high enough (Yang et al., 2022b).

### 4.8 Maillard reaction

Glycosylation technology is widely used to modify proteins. This method involves the covalent bonding of carbohydrates to proteins through the Maillard reaction. This results in the creation of conjugates that can improve the functional properties of the proteins, such as their solubility, emulsification, and water absorption properties (Priola and Lawson, 2001; Wang et al., 2020c).

To date, only a few studies have focused on the modification of quinoa protein via glycosylation technology. Teng et al. (2021) conducted a study in which they modified quinoa protein isolate by reacting it with either mannose or xylose at 60 °C at different ratios. They used SDS‒PAGE analysis to monitor structural changes and observed protein band intensity changes and movement. After the Maillard reaction, the glycosylated quinoa protein isolate demonstrated improved solubility, emulsifying properties, and water and oil absorption capacity.

### 4.9 Enzymatic modification

Limited enzymatic hydrolysis is a process in which enzymes are used to breakdown proteins into smaller peptides or amino acids. Enzymatic hydrolysis is often used in the food industry to modify the properties of proteins to improve their performance in various applications. It can change the functional properties of the protein, such as its solubility, gelation property, and the ability to foam or emulsify. It can also be used to alter the nutritional properties of the protein, such as increasing its digestibility or bioavailability.

Alcalase® has been used to hydrolyse quinoa protein (Aluko and Monu, 2003; Wang et al., 2022). These studies found that limited alcalase® hydrolysis was able to alter the gelling properties, solubility, emulsifying properties and foaming properties. Limited alcalase® hydrolysis affected the secondary structure of quinoa protein and intermolecular hydrogen bonding between polypeptides, leading to an improvement in the gel strength of quinoa protein isolate hydrolysates (Wang et al., 2022). On the other hand, protein hydrolysis resulted in the dissociation of insoluble protein aggregates into smaller peptides with increased exposure of hydrophilic groups, which could explain the better solubility than the native protein concentration (Aluko and Monu, 2003). However, quinoa protein hydrolysates from protease showed negative effects on gelling properties with a less interconnected protein network, which was attributed to the decreased surface hydrophobicity of the protein samples (Galante et al., 2020). In addition, quinoa protein hydrolysates from pancreatic enzymes composed of amylase, trypsin, lipase, ribonuclease, and protease were evaluated by Daliri et al. (2021). Pancreatin hydrolysate of quinoa protein concentrate showed higher solubility, emulsifying and foaming activities but lower emulsifying and foaming stabilities than that of the control. The high emulsifying/foaming activity but low stability may be because the low molecular weight peptides did not have sufficient amphiphilic and superficial characteristics to stabilize emulsions or foam systems due to their short chains (Daliri et al., 2021). In conclusion, several studies have confirmed that enzymatic hydrolysis can be an option to modify the functional properties of quinoa protein to fulfil the needs of the food industry.

## 5 Digestibility of quinoa protein

When proteins are consumed by humans, they are broken down into short polypeptides and amino acids with varying molecular weights by enzymes in the stomach and small intestine. Several factors affect protein digestibility, including the protein's native structure, processing conditions, and food ingredient interactions. The digestion of proteins starts from the stomach with the help of protease pepsin in the gastric fluid into broken peptides. In addition, gastric acid facilitates protein digestion by unfolding proteins, so proteases have better access to the cleavage sites. Then the peptides proceed to the small intestine where pancreatic proteases work to further cleave undigested substances into oligopeptides and amino acids. Various transport proteins subsequently move the amino acids via the mucosal cells into blood and eventually the amino acids go to the liver (Goodman, 2010). However, proteins may remain incompletely digested and reach the colon where they are metabolized by colonic microflora (Kaur et al., 2022). The metabolites produced during the digestion of proteins can have negative impacts on colonic health. This can include conditions such as colorectal cancer and inflammatory bowel disease. The severity of these impacts depends on the rate at which toxic metabolites are generated and how well the body can detoxify and excrete them from the large intestine (Peled and Livney, 2021). Therefore, proteins with high digestibility are favoured to achieve better health outcomes from a nutritional point of view.

The protein digestibility corrected amino acid score (PDCAAS) is used to evaluate protein quality based on the comparison of the essential amino acid content of a test protein with that of a reference essential amino acid pattern and a correction for differences in protein digestibility as determined using a rat assay (Schaafsma, 2005). The digestible indispensable amino acid score (DIAAS) was introduced by the FAO in 2013 as a way to more accurately measure protein digestibility. It is based on ileal digestibility, rather than faecal digestibility, because endogenous amino acid losses may occur at the end of the ileum due to antinutritional factors (Kaur et al., 2022). Quinoa protein was reported to have a PDCAAS of 0.68 (Scanlin and Lewis, 2017). The authors suggested that Maillard reaction products during drum drying and damage to lysine during heat treatment during the processing of quinoa proteins probably lowered the PDCAAS score (Scanlin and Lewis, 2017). To the authors’ knowledge, there is no reported DIAAS score for quinoa protein. Elsohaimy et al. (2015) revealed that the *in vitro* digestibility of quinoa protein isolate was 78.37 ± 1.08%, which was in line with the previous measurements by Repo-Carrasco-Valencia and Serna (2011) (76.3–80.5%) and Nasir et al. (2015) (75.95 ± 0.29 to 78.11 ± 0.43%). These data indicated that quinoa protein had equivalent digestibility to other plant proteins, such as rice, corn, and beans, but its digestibility was lower than that of animal proteins (Nasir et al., 2015; Shi et al., 2020).

Several methods have been employed to process plant proteins to achieve the desired digestibility by altering their structures (i.e., unfolding, crosslinking and aggregation). The protein’s response to these processing treatments can affect both its digestibility and the rates of releasing polypeptides or amino acids. Current methods to alter quinoa protein digestibility mainly focus on heat treatments. Ruales and Nair (1994) investigated the *in vitro* digestibility of quinoa protein using different processing methods, including cooking, autoclaving, drum‒drying and extrusion. Prolonged cooking for 60 min reduced the protein digestibility mainly because of protein denaturation. Autoclaved and extruded samples showed higher protein digestibility. Precooking quinoa protein before drum‒drying led to a slight decrease in digestibility due to the Maillard reaction between increasing reducing sugars and lysine residues in the protein (Ruales and Nair, 1994). Additionally, these authors demonstrated the detrimental effects of saponins on quinoa protein digestibility. The effect of extrusion on quinoa protein digestibility was also reported by Muñoz-Pabon et al. (2022), indicating that extruded quinoa flour products showed significantly higher *in vitro* protein digestibility than raw quinoa flour. This was attributed to the high extrusion temperature (105 °C), which can alter the secondary structure of the quinoa protein, making it more soluble and more susceptible to digestive enzymes (Dakhili et al., 2019; Muñoz-Pabon et al., 2022). Furthermore, gun puffing and extrusion puffing yielded high‒quality puffed quinoa grains with enhanced *in vitro* protein digestibility (93.4% and 87.2%, respectively) (Zapana et al., 2019). Moreover, different heating methods could have opposite impacts on the *in vitro* digestibility of quinoa protein. For instance, Cao et al. (2023) applied a water bath (70–90 °C for 30 min) and microwave (70–90 °C for 30 min) to quinoa protein suspensions to form quinoa protein gel and investigated the digestibility of quinoa protein gel prepared by different methods. The results showed that the digestibility of the quinoa protein gel prepared in a water bath increased from 65.55 to 75.68% as the temperature increased from 70 °C to 90 °C, while the digestibility of the gel prepared in a microwave decreased from 81.40% to 71.87% as temperature increased from 70 °C to 90 °C. This finding could be attributed to the various gel networks derived from different processing methods, where heating in a water bath led to small and loose aggregation networks, while microwave treatment promoted a compact and orderly gel network which makes it more difficult for proteases to access and hydrolyse the protein (Cao et al., 2023).

The *in vitro* digestibility of quinoa protein under heat processing treatment was strongly influenced by processing temperature, protein extraction conditions, and other grain components (Ruiz et al., 2016a). An increase in temperature or extraction pH led to protein denaturation and aggregation, leading to decreased digestibility. Moreover, quinoa protein isolates had higher digestibility than wholemeal quinoa flour, indicating that the presence of other components (mainly starch, fibre, and fat) may have an interactive impact that hinders enzymatic hydrolysis at high temperatures (90–120 °C) (Ruiz et al., 2016a).

In addition to heat treatments, fermentation was applied to quinoa protein concentration and improved its digestibility. Alrosan et al. (2023) reported an improvement in the *in vitro* digestibility of quinoa protein from 78.5% to 87.7% after 5 days of fermentation with kefir grains that was correlated with changes in the secondary protein structure. Proteolysis of quinoa protein by *Lactobacillus* during fermentation played an important role in promoting protein digestibility, as proteins were hydrolysed into oligopeptides and eventually amino acids. In addition, FTIR results showed a significant increase in random coil content and a decrease in α‒helix content in the secondary protein structure during the fermentation process, where a decrease in α‒helix content was highly correlated with the enhanced protein digestibility (Alrosan et al., 2023).

## 6 Applications

Quinoa has gained much attention for its high protein content and exceptional nutrient profile. As interest in plant‒based proteins continues to grow and more efforts are spent on the modification of quinoa protein functionality, the application of quinoa protein in various industries has also seen a significant increase. From food and beverage products to cosmetics and personal care items, the versatility of quinoa protein makes it a valuable ingredient with a wide range of potential uses.

The current food applications of quinoa mainly focus on gluten‒free foods for celiac disease patients such as bread (Jagelaviciute and Cizeikiene, 2021), biscuits (Sandez Penidez et al., 2022), snacks (Muñoz-Pabon et al., 2022; Zapana et al., 2019) and crackers (Meriles et al., 2022). These applications used quinoa flour as the raw material for replacing a part of traditional cereals. The results showed that quinoa is a competitive candidate for use as a fortification and supplementation in food. The applications also involve pasta (Gupta et al., 2021), sausages (Fernández-López et al., 2021), and beverages (Lorusso et al., 2018; Pineli et al., 2015). A sausage‒like gel developed from quinoa flour was characterized by Felix et al. (2021), indicating the potential of quinoa flour for meat‒analogue products. These applications indicate that quinoa protein has suitable functional properties, especially emulsifying properties and gelling properties, and that it might be possible to improve these properties through various modifications. In addition, due to the superior nutritional value of quinoa protein, it was suggested that quinoa protein isolates could be introduced into infant formula (Venlet et al., 2021). Moreover, quinoa protein is promising in building delivery systems, and the encapsulation of flavonoids, polyphenols and essential oils has been reported by Liu et al., 2021, Liu et al., 2022 and Chen et al. (2023).

## 7 Conclusion and future research

Overall, quinoa protein possesses several advantages over many plant proteins, including a well‒balanced amino acid profile, high digestibility, and gluten‒free features. This review highlights the impacts of modification on the functional properties and *in vitro* digestibility of quinoa protein. The enhanced properties via various modifications made quinoa protein a competitive candidate for replacing animal protein to fulfil customers’ needs. Among all the functional properties, good emulsifying properties and gelling properties support quinoa protein as a promising option for novel food production. Therefore, quinoa protein has been employed in various applications from gluten‒free foods, meat analogues, and beverages to delivery systems and cosmetics.

To widen the application of quinoa protein, future research will aim to improve the production efficiency and scalability of quinoa protein isolate. This may include studying processing techniques and process simplification to increase the yield and purity of the protein. Additionally, new modification methods such as high/low moisture extrusion, shear cell processing and ultrahigh‒pressure processing could be investigated to improve the digestibility and functional properties of quinoa protein. Another area of focus will be developing new food applications for quinoa protein, such as plant‒based alternatives to animal‒based products (e.g. meat analogues), as well as incorporating plant protein into a wide range of food products for functional and nutritional benefits.

## Funding

The research was supported by the 10.13039/501100003524Ministry of Business, Innovation and Employment (MBIE) as part of the New Zealand‒China Strategic Research Alliance (10.13039/100008709SRA) project.

## CRediT authorship contribution statement

**Hao Cui:** Writing – original draft, Methodology, Investigation. **Siqi Li:** Writing – review & editing, Supervision. **Debashree Roy:** Writing – review & editing, Supervision. **Qing Guo:** Supervision, Writing – review & editing. **Aiqian Ye:** Conceptualization, Funding acquisition, Writing – review & editing, Supervision.

## Declaration of competing interest

The authors declare that they have no known competing financial interests or personal relationships that could have appeared to influence the work reported in this paper.

## Data Availability

No data was used for the research described in the article.
